# Radiology Staff Experiences With Integrating Artificial Intelligence Into Radiology Practice in a Swedish Hospital: Qualitative Case Study

**DOI:** 10.2196/77843

**Published:** 2025-12-22

**Authors:** Per Nilsen, Petra Svedberg, Ingrid Larsson, Lena Petersson, Jens Nygren, Emilie Steerling, Margit Neher

**Affiliations:** 1 School of Health and Welfare, Halmstad University, Box 823 Halmstad, Halmstad, 30118, Sweden, 46 0706341151; 2 Department of Health, Medicine and Caring Sciences, Linköping University, Linköping, Sweden

**Keywords:** radiology, health care, staff, artificial intelligence, AI, integration, case study

## Abstract

**Background:**

The integration of artificial intelligence (AI) in radiology has advanced significantly, but research on how it affects the daily work of radiology staff is limited.

**Objective:**

This study aimed to explore the experiences of radiology staff on the integration of an AI application in a radiology department in Sweden. This understanding is essential for developing strategies to address potential challenges in AI integration and optimize the use of AI applications in radiology practice.

**Methods:**

This qualitative case study was conducted in a single radiology department with 40 employees in 1 hospital in southwestern Sweden. The study concerned the integration of an AI-powered medical imaging software designed to assist radiologists in analyzing and interpreting medical images. Using a qualitative design, interviews were conducted with 7 radiologists (physicians), 4 radiologic technologists, and 1 physician assistant. Their experience within radiology varied between 13 months and 38 years. The data were analyzed using qualitative content analysis.

**Results:**

Participants cited numerous strengths and advantages of significant value in integrating AI into radiology practice. Numerous challenges were also revealed in terms of difficulties associated with choosing, acquiring, and deploying the AI application and operational issues in radiology practice. They discussed experiences with diverse strategies to facilitate the integration of AI in radiology and to address various challenges and problems.

**Conclusions:**

The findings illustrate how AI integration was experienced in 1 hospital. While not generalizable, the study provides insights that may be useful for similar settings. Radiology staff believed AI integration enhanced decision-making and quality of care, but they encountered challenges from preadoption to routine use of AI in radiology practice. Strategies such as internal training and workflow adaptation facilitated the successful integration of AI in radiology.

## Introduction

The integration of artificial intelligence (AI) applications into radiology practice has advanced significantly, offering promising solutions to enhance diagnostic accuracy, improve workflow efficiency, and, ultimately, optimize patient care [1]. Traditionally, radiologists (ie, medical doctors specialized in diagnosing and treating injuries and diseases using medical imaging procedures) have relied on their expertise and visual interpretation skills to analyze medical images. However, the increasing complexity and volume of imaging studies, coupled with increasing demands for faster and more accurate diagnoses, have underscored the need for innovative solutions to support radiology staff [2-4].

AI in radiology practice has a wide range of applications, from image acquisition, processing, and interpretation to aided reporting, follow-up, planning, data storage, and data mining [5]. Enhanced diagnostic efficiency has been demonstrated with AI applications within radiology across various imaging modalities, including radiography, computed tomography (CT), magnetic resonance imaging (MRI), and mammography [1,6]. Furthermore, AI applications have been shown to streamline radiology workflow by automating routine tasks, improving productivity, and reducing turnaround times for image interpretation and reporting [7-9].

The impact of AI applications in radiology has been discussed extensively in opinion articles [10-16], and various practical use cases have been described in the literature [17-22]. There are also clinical trials assessing the efficiency of AI applications in improving diagnostic accuracy [1,23,24]. In addition, surveys have explored radiologists’ perceptions and expectations regarding AI use in their field [25-30].

However, there is a gap in research addressing how real-world use of AI applications in radiology practice affects the daily work of radiology staff. Studies are also lacking with regard to procuring and deploying AI applications, as well as firsthand experiences of staff in radiology departments. Thus, there are gaps in understanding the practical implications of AI integration in this field.

Given that little is known about the everyday use of AI in radiology, a case study approach allows an in-depth exploration of how integration was experienced in a particular organizational context. The aim of this study was to explore the experiences of radiology staff on the integration of an AI application in a radiology department in Sweden. The study sought to provide insights into the real-world application of AI in radiology, with a particular focus on understanding the practical implications of AI integration based on the experiences of staff in a radiology department. This understanding is essential for developing strategies to address potential challenges in AI integration and optimize the use of AI applications in radiology practice.

## Methods

### Study Design and Setting

We adopted a qualitative case study design [31], using an inductive qualitative content analysis [32] to examine the integration of an AI application in a radiology department of 1 Swedish hospital. The study adhered to the COREQ (Consolidated Criteria for Reporting Qualitative Research) to ensure trustworthiness [33].

The study setting was 1 radiology department (with 40 employees) in 1 hospital with approximately 4000 employees in southwestern Sweden. Radiology departments in Swedish hospitals typically serve primary care and other wards within the hospitals, including emergency departments. This hospital was selected due to its active integration of AI applications in radiology, providing a relevant and practical setting for exploring real-world experiences with AI integration. The radiology department had prior exposure to AI-assisted workflows, making it a suitable case for examining both the benefits and challenges of implementation.

Health care in Sweden is organized at the regional level. Each of the country’s 21 regions is responsible for providing health care services to its residents. The regions are responsible for managing and funding health care services, including hospitals, primary care, and specialized health care services. The health care system is funded through taxes and is primarily publicly funded; private health care providers also play a role. Patients have the right to choose their health care service provider and have access to a wide range of services, including preventive care, medical treatment, and rehabilitation services.

### The AI Application

The study examined the integration of AI-Doc, an advanced AI-powered application specifically designed to enhance radiology practice by augmenting the capabilities of radiology staff, improving diagnostic precision, and optimizing workflow efficiency. AI-Doc leverages machine learning algorithms and deep learning techniques to process and analyze a diverse range of imaging modalities, including radiographs, CT scans, MRI, and ultrasound images.

The application autonomously identifies anomalies, highlights areas of potential concern, and generates preliminary diagnostic insights. Acting as an “intelligent assistant,” AI-Doc reduces cognitive strain on radiologists while promoting greater diagnostic consistency and accuracy across cases. Its functionality includes customizable alert systems, integration with picture archiving and communication systems, and a user-friendly interface designed to blend into radiologists’ existing workflows.

AI-Doc has seen widespread adoption in Swedish health care systems, where it serves as a practical, real-world example of how AI technology can be integrated into modern radiology. The application has been described and methodically evaluated in the work of Wiklund and Medson [34], where its impact on clinical workflows and diagnostic outcomes was further assessed.

### Study Participants and Recruitment

The practice context for using the AI application was identified through the network of the research group. The head of the radiology department at the hospital was contacted by the research team and informed about the study. The head assisted by supplying the email addresses of relevant employees in the radiology department. Eligibility criteria were that participants were radiology staff who were directly engaged in the work with the AI application and the work routine around the application at the radiology department.

Participants signed up by responding to an email invitation with detailed information about the study. Two reminders were sent at 2-week intervals. The invitation message clearly stated that participation was voluntary and that participants could withdraw from the study at any time and without explanation.

Participants were recruited from diverse professional groups to achieve a varied sample and a broad perspective on the integration of the AI application. The study population consisted of 12 staff members from the radiology department: 7 radiologists (physicians), 4 radiologic technologists (referred to as radiographers outside North America), and 1 physician assistant. Seven were men, and 5 were women. Their experience within radiology varied between 13 months and 38 years.

Participants were selected based on their direct engagement with the AI application and their involvement in related workflows within the radiology department. The study aimed to capture a diverse range of perspectives, which is why radiologists, radiologic technologists, and a physician assistant were included. Radiologists were selected for their expertise in interpreting medical images and their decision-making role in integrating AI into clinical practice. Radiologic technologists were included due to their hands-on role in image acquisition and processing. The physician assistant was selected to provide insight into how AI influences patient interactions and radiology workflow coordination. This diverse sample allowed for a comprehensive exploration of AI integration from multiple professional viewpoints.

### Data Collection

Semistructured interviews were conducted online via Zoom or Teams by 3 of the co-authors (ES, MN, and LP) between September 2023 and May 2024. Two pilot interviews were performed to test the interview guide. No adjustments to the questions were made, and these 2 interviews were therefore included in the study. The authors had no preexisting relationship with any of the participants. Before starting the interviews, the participants were asked if they had read the information about the study and to give their written informed consent to participate.

An interview guide, consisting of open-ended questions, directed the course of the interviews. The interview guide included questions on the participants’ experiences regarding the integration of the AI application, encompassing everything from selecting and purchasing the application to using it in routine practice. Questions also addressed the impact of the AI application on professional work roles and participants’ trust in the AI application. Probing questions were asked during the interviews to clarify the description or draw attention back to the topic; for example, “What do you mean?” and “Can you explain this a little further?”

The interview guide ensured that all participants were asked similar core questions while allowing flexibility for follow-up prompts to capture nuanced insights. The research team maintained regular discussions throughout the data collection process to align their approach and ensure uniformity in questioning. Furthermore, investigator triangulation was applied during data analysis, with multiple researchers independently reviewing transcripts to enhance reliability and minimize individual biases.

Interviews lasted for an average of 45 minutes (SD 8.02, ranging from 24 to 55 min). Interview data were audio recorded and transcribed verbatim.

### Data Analysis

The data were analyzed using qualitative content analysis, following the approach for conventional content analysis as outlined by Hsieh and Shannon [32]. This data-driven analysis focused on the participants’ unique experiences, rather than being guided by a preexisting theory or hypothesis. Investigator triangulation was used to enhance the validity of the findings. Three of the researchers (PN, MN, and ES) independently reviewed and reread the transcripts to develop a comprehensive understanding of the content and gain an overview of the material.

With the study’s aim in focus, the first author (PN) highlighted relevant text and made margin notes to capture all key aspects of the content. Initial thoughts and impressions were documented during this process. He then generated codes from the data to reflect the most significant content. Related codes were then grouped and organized into subcategories and broader categories. These categories and subcategories were then presented to the research team, which led to an iterative process that involved continually revisiting and refining the codes, ensuring they were consistent with the full scope of the material.

The subcategories and categories were then systematically compared for differences and similarities to ensure they were as internally consistent and externally distinct as possible. Labels were assigned to each category and subcategory, and numerous revisions were made to ensure they reflected the content accurately. The names and content of these labels were discussed thoroughly among the research team, and adjustments were made until consensus was reached. In the next step, the first author (PN) selected representative quotes from the interviews to illustrate each subcategory. After the team discussion, the chosen quotes were translated into English by PN.

### Ethical Considerations

Ethical approval was granted by the Swedish Ethical Review Authority (no. 2023‐05318), and the study was conducted according to the World Medical Association Declaration of Helsinki ethical principles for medical research involving human subjects [35]. Participants were assured that their data and their personal integrity would be safeguarded throughout the research process and that they would not be individually recognizable in the final scientific publication. They were asked to sign a formal consent document before the interview and informed that they were free to withdraw at any time, no questions asked. Participants consented to the publication of the quotes.

## Results

### Overview

**Figure 1.**
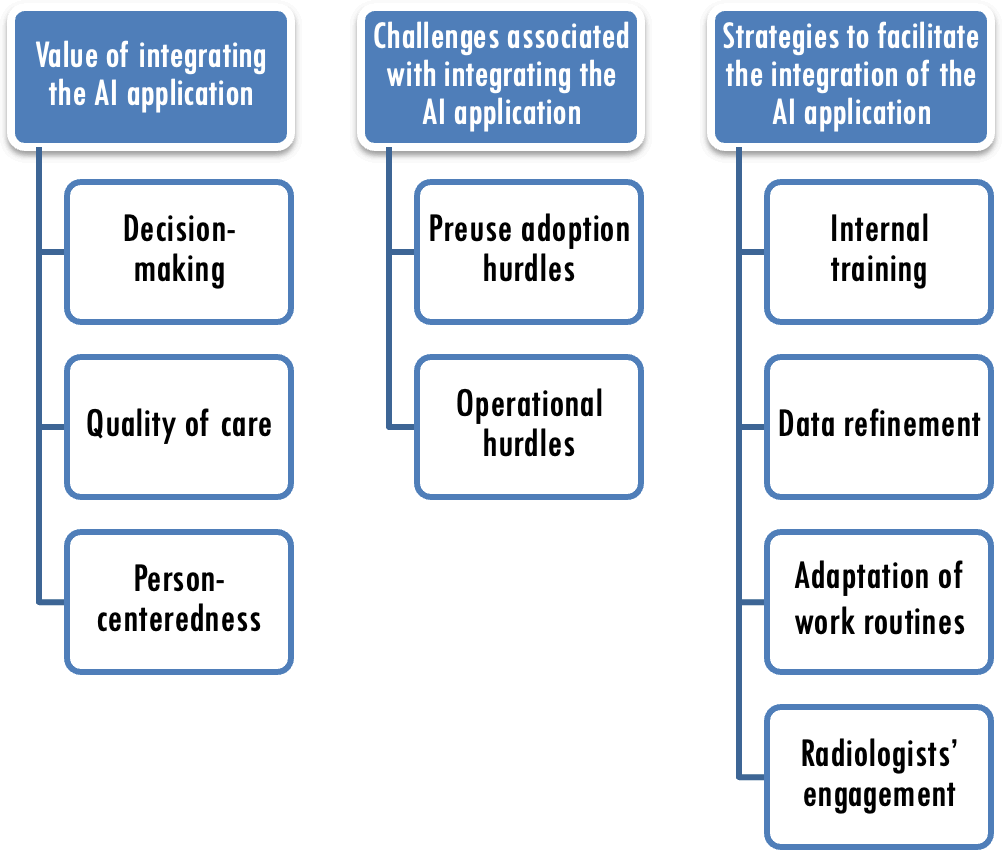
Three categories and 9 subcategories encapsulate the participants’ experiences associated with integrating the AI application into radiology practice. AI: artificial intelligence.

Three categories and 9 subcategories emerged from the analysis of the interview data (Figure 1). These categories and subcategories encapsulate the participants’ experiences associated with integrating the AI application into radiology practice.

### Value of Integrating the AI Application Into Radiology Practice

#### Overview

Participants mentioned several areas where they experienced significant value in using AI in radiology practice, citing numerous strengths and advantages they experienced. This value proposition was delineated into three subcategories: decision-making, quality of care, and person-centeredness.

#### Decision-Making

Participants experienced that the AI application augmented their decision-making capabilities by providing better image analysis because the application identified details that were invisible to the human eye. This was experienced as facilitating more accurate diagnoses and fostering confidence in clinical decisions.

The AI system relieves us as radiologists from something that we find boring and time-consuming. And which we might also feel a little unsure about because it’s easy to miss something.

[#6, radiologist]

The AI system has very high accuracy. It is very rare for it to flag a false finding.

[#8, radiologic technologist]

The AI application was particularly important for junior radiologists who were less experienced in analyzing images. Participants believed the AI application refined their skills because it provided detailed analyses of imaging scans. This helped them identify subtle patterns they might otherwise have missed. They experienced that this exposure sharpened their diagnostic skills.

The AI system has the potential to save some time and to make one feel more confident in the response, especially as a newcomer in the profession.

[#4, radiologist]

#### Quality of Care

Participants also reported experiences of how the integration of the AI application into radiology practice could benefit the overall quality of care. They experienced that the AI application promoted consistency by applying uniform diagnostic criteria across all cases, reducing variability in image interpretation and supporting standardized decision-making for more reliable diagnoses. This, in turn, enhanced equity in health care delivery by ensuring that all patients received the same high-quality diagnostic care, regardless of geographic location, hospital resources, or clinician expertise.

Knowledge will be more equitable with AI.

[#1, radiologist]

The AI system will primarily increase accessibility [to healthcare].

[#8, radiologic technologist]

There will be a more standardized way of working with AI.

[#9, radiologic technologist]

Participants lauded the AI application for its effectiveness in detecting incidental findings because it assisted them in identifying anomalies. This capability enabled early intervention, potentially leading to better patient outcomes.

And the AI system really removes the risk for patients of developing symptoms from these clots.

[#11, radiologic technologist]

Lung embolisms are detected that one might not know about if they hadn’t been reviewed using the AI system.

[#12, physician assistant]

#### Person-Centeredness

Participants experienced that the integration of the AI application into radiology practice had a positive impact on person-centeredness in terms of considering the whole person and accounting for the individual needs and preferences of patients. They highlighted enhanced accessibility for patients to radiologic examinations and diagnostic procedures. Participants emphasized that the AI application shortened the duration of patients’ health care interactions by streamlining the examinations and reducing wait times for diagnostic results.

With these image enhancements, the patient is exposed to a lower dose of radiation. … The patient receives a faster diagnosis.

[#2, radiologist]

They can receive a response immediately. They might live in Ullared, and previously they would go home and then be called back here again. This improves things for our patients in that way, it really does.

[#12, physician assistant]

In addition, participants experienced that the AI application played a vital role in reassuring many patients. The application generated images that patients could access after their examination, fostering trust in the technology to minimize errors and enhance the accuracy of their diagnoses.

Many patients are not satisfied with the doctor saying that this is a herniated disc and you just have to go to rehab. Instead, they want an image of things. They want proof.

[#8, radiologic technologist]

### Challenges Associated With Integrating the AI Application Into Radiology Practice

#### Overview

Participants experienced numerous challenges in terms of difficulties or problems associated with choosing, acquiring, and deploying the AI application and its subsequent use in radiology practice. These challenges were classified into two subcategories: preuse adoption hurdles and operational hurdles.

#### Preuse Adoption Hurdles

Participants described several types of challenges associated with procuring and setting up the AI application in radiology practice. These challenges were experienced in the preuse adoption phase, that is, before the application was integrated into radiology practice. They underscored the significant financial investment they associated with AI applications.

These are quite expensive things. We're talking about millions, of course, for licenses and hardware and so on.

[#9, radiologic technologist]

Navigating the crowded marketplace of AI applications posed a considerable challenge in identifying and selecting the optimal solution for radiology departments.

There are hundreds of products on the market.

[#2, radiologist]

Participants complained about the protracted nature of the adoption process for the AI application, which entailed addressing numerous administrative hurdles. Furthermore, the actual deployment of the AI application was marked by technical intricacies.

It seems to be a huge hurdle for many places, and IT puts a stop to it. They say it’s too complicated to implement an AI. It can take a year from asking questions until you get a final answer.

[#1, radiologist]

This implementation of the [AI application] takes an incredibly long time and is so slow.

[#7, radiologist]

#### Operational Hurdles

Participants experienced numerous practical operational difficulties that could arise in the everyday use of the AI application. They emphasized the steep learning curve that radiology staff must traverse to harness the application’s full potential. Navigating technical issues inherent to the application provided a challenge.

Unfortunately, it quite often freezes or lags, or you get logged out. Or some sort of system error. Or some local [error].

[#3, radiologist]

It takes a long time to program. One needs to have time for testing, so it often actually takes a year before everything is set up as desired. Because there is so much to understand when you get a completely new machine.

[#8, radiologic technologist]

Participants also experienced that data management could provide obstacles for the effective integration of the AI application into radiology practice.

It is very important how all the data management is handled. Information is first sent to a local server where the data is anonymized before it is sent to a cloud solution, and then it bounces back. [#2, radiologist]

### Strategies to Facilitate the Integration of the AI Application Into Radiology Practice

#### Overview

Participants highlighted experiences of using diverse strategies to facilitate the integration of the AI application into radiology practice to address various challenges and problems they encountered. These strategies encompassed both specific actions tailored to support AI use and the active involvement of individuals taking initiatives to facilitate the use. Four subcategories emerged from the data analysis: internal training, data refinement, adaptation of work routines, and radiologists’ engagement.

#### Internal Training

Participants emphasized the importance of in-house, internal training, which consisted of educational activities conducted within the health care organization’s premises or by its staff. This type of training was designed to educate employees or team members about the specific AI application in radiology.

Training is required for all users to understand what the [AI system] is, what the tool is supposed to do, in what context it will be used, and why it exists.

[#1, radiologist]

Training sessions [are needed] as well, of course. As always when introducing new things, someone needs to present it. … present the applications, how they look, and what buttons to press.

[#6, radiologist]

The relevance of integrated training to support the integration of the AI application into radiology practice was also stressed by participants. This training could be included as part of the overall acquisition of the AI application, which meant that the health care organization received both the AI application and the necessary training to ensure that the radiology staff would have the knowledge and skills to leverage the AI application effectively.

You can also have someone from the company [that sold the AI system] come and help you, explaining how to use the system and if there are any pitfalls with this new technology. ... The purchase usually comes with a certain number of hours of support.

[#8, radiologic technologist]

There are many lectures [on AI] that one can attend. ... And then there was a guy who had a lecture in the main hall about AI, so there is a great interest.

[#12, physician assistant]

More informal training also occurred in the use of the AI application through everyday conversations and discussions with colleagues who helped each other.

I do my review in parallel [with the AI system], and if it matches, you gain trust.

[#3, radiologist]

Well, there were colleagues who, in situations [when problems arise], when it... I mean, there is so much to learn. ... Then it becomes a brief discussion with a specialist who has more experience regarding how it works.

[#7, radiologist]

#### Data Refinement

The importance of ensuring local validation and adaptation of data was emphasized by participants for the integration of the AI application into radiology practice. These were iterative processes intended to improve and optimize the AI application to ensure its accuracy, reliability, and effectiveness. Validation was a process of verifying the performance and accuracy of the AI application using data that were representative of the local environment.

Even before the AI system was implemented, a validation study was conducted where they reviewed a hundred or up to a thousand cases to see how the AI solution performed on our patient population, our machines, our settings, and so on. It is important to carry out such a validation process.

[#2, radiologist]

We placed quite a bit of emphasis on the configuration from the start, ensuring that the AI system would assist us; we were careful to get the setup right from the very beginning.

[#10, radiologic technologist]

#### Adaptation of Work Routines

Some changes in the radiology staff’s current work processes and patient pathways were necessary to facilitate the integration of the AI application into radiology practice. This involved partial adjustment of both radiologists’ and radiologic technologists’ work routines to align with a revised sequence of actions for radiology patients. The participants experienced these modifications as essential to enable patients to leverage the advantages of the AI application fully.

We had to find some form of structure for who would be responsible when a study needs to be followed up. ... The AI system requires some adjustments to the routine.

[#5, radiologist]

The working method has changed. Patients stay a bit longer and receive information before they leave. The new routine gives [us radiologic technologists] more contact with the radiologists.

[#11, radiologic technologist]

#### Radiologists’ Engagement

Individual radiologists played a significant role by functioning as influential change agents to facilitate the use of the AI application in radiology. Participants experienced that these individuals advocated for the benefits of AI and communicated its value to colleagues and managers in radiology and the wider health care organization.

It is not something that has come from outside; rather, the AI issue is something that we in the medical group have driven. We are positive, and these are our tools.

[#6, radiologist]

There was a sense of security from the very beginning because doctors were involved in the implementation of the AI system.

[#11, radiologic technologist]

The radiologists addressed resistance and skepticism among their colleagues toward AI by engaging in discussions about the application and addressing various concerns. Furthermore, they organized training and helped colleagues understand how the AI application worked and how to use it most effectively.

I was one of the “super users” because I work with this daily. I was present to answer questions so that we could have ongoing discussions about whether there was anything that needed to change in how the tool looked or was presented. Then we could communicate that to the AI company.

[#1, radiologist]

We have a doctor who is quite involved in this. He has also given lectures on it. He has promoted the AI system to his colleagues and talked about it.

[#10, radiologic technologist]

## Discussion

### Principal Findings

On the basis of interviews with radiologists, radiologic technologists, and a physician assistant in 1 radiology department in a Swedish hospital, this qualitative case study sought to explore experiences concerning the integration of an AI application into radiology practice, from purchasing to using the app. The participants reported that the integration brought significant benefits, particularly in terms of improved diagnostic accuracy, quality of care, and person-centeredness. However, they also identified challenges they experienced, both preuse adoption hurdles and operational hurdles. Despite these challenges, the participants experienced that strategies such as internal training, data refinement, adaptation of work routines, and radiologists’ engagement could facilitate the successful integration of AI into radiology practice. While the findings are not intended to be generalizable, they offer transferable insights that may inform AI integration efforts in similar health care contexts.

The participants experienced numerous benefits that demonstrated the substantial value of integrating the AI application into their radiology practice, citing advantages for themselves, the health care system, and patient care. Compared with other areas of health care, where AI integration has been slower, AI in radiology is well-established, optimized by the extensive digitization of imaging data that has been underway since the late 20th century [30]. This transition began in the 1970s with the introduction of CT scans, which used x-rays and computer processing to produce digital images. The advent of MRI in the 1980s further advanced this digital shift, and by the 1990s, most radiology departments had moved from film-based to digital imaging. This evolution has made AI a mature and indispensable technology in radiology, proving its significant value in the field.

Although the significance of integrating AI applications into radiology is widely recognized, research has shed light on numerous challenges associated with this use, many of which we also observed. Previous studies on AI use in radiology [28,36,37] have identified technical obstacles related to data quality, standardization, transparency, interpretability, and regulatory compliance. Ethical concerns concerning data privacy and security are also critical factors that need to be addressed to promote the ethical and responsible use of AI in radiology [19,38,39]. While our findings resonate with the broader literature, they reflect the specific circumstances of 1 radiology department in a Swedish hospital and thus should be understood as context bound.

The participants in our study highlighted many strategies to facilitate the successful integration of the AI application into radiology practice. It required both formal learning based on organized training and informal learning that was more spontaneous. Informal learning refers to the acquisition of knowledge and skills that takes place outside of a structured, formal educational environment [40]. This learning in our study seemed to happen through everyday interactions and activities, which is typical for informal learning because it occurs without a specific curriculum or defined objectives. Informal learning is typically self-directed, voluntary, and motivated by the learner’s interests or needs [41]. In our study, this learning was driven by the radiology staff’s ambition to solve problems that occurred when using the application. Learning by doing seemed to be very important for the participants in our study. In this form of learning, individuals acquire knowledge and skills through direct experience and active participation in real-life tasks or activities [42-44].

Successful integration of the AI application into radiology practice in our study also depended on local validation and adaptation of data as well as adaptation of existing routines around the application. The findings suggest that the use of AI in radiology can have ripple effects, that is, indirect or unintended consequences that result from introducing a new practice within a health care system. Participants described that they had established new workflows and patient pathways. Ripple effects can have both positive and negative impacts on various aspects of health care delivery and patient care [45]. In our study, these effects were largely experienced as positive, notably by shortening the duration of patients’ health care interactions through streamlined examinations and reduced wait times for diagnostic results. Ripple effects are often underestimated when implementing new practices in health care due to the complexity and interconnectedness of health care systems. Various stakeholders, structures, and processes can be affected by changes, making it challenging to anticipate all potential impacts [46,47].

The importance of engagement by individual radiologists throughout the process of procuring, deploying, and using the AI application in radiology was evident from the interviews in our study. Radiologists are usually highly respected and influential within health care; colleagues and patients typically trust radiologists due to their extensive education, training, and experience [48]. Our findings showed that radiologists’ recommendations and opinions regarding the AI application held significant weight. Some radiologists essentially functioned as change agents as they promoted the use of the application to colleagues. Although there are several terms for change agents in the literature, including champions, facilitators, and super users, there is consensus that change agents can play a crucial role in influencing others to adopt and implement new ideas, products, or practices by providing information, support, and encouragement [49,50].

Despite the positive experiences of the participants in our study concerning the integration of the AI application into radiology practice, research has revealed apprehension among radiologists about the automation of tasks traditionally performed by human experts [28,51-53]. Uncertainty about the future of the profession has been observed to have an impact on radiology students, who are hesitant about committing to a career path that seems susceptible to technological disruption [14]. There is also a debate that many traditional tasks will increasingly be taken over by AI, with the result that radiology staff (and health care staff in general) will need to spend less time on routine tasks, which may result in a decline in some clinical skills [54]. Deskilling is not a new phenomenon; however, technological advancements have reduced skill requirements for many jobs in various societal sectors [55]. The question is how or to what extent it may occur in radiology, a topic that warrants further investigation.

Another contemporary debate about AI is its “black box” nature, which refers to the difficulty in interpreting or explaining the reasoning behind specific outputs due to the inherent complexity of AI applications, both in how they are developed and trained and in how they are applied [56]. However, in our study, this appeared to be relatively unimportant for the participants, who displayed a high level of trust in the AI application they used. Their trust seemed to be built up over time through consistent and reliable performance of the application. As they worked alongside the application, they were able to see firsthand how the technology could aid in interpreting images accurately and efficiently. Research suggests that the need for explainability diminishes when AI consistently outperforms human practitioners [57]. Thus, trust in AI appears to be based on the reliability of the outputs rather than the transparency of the processes.

### Strengths and Limitations

Several limitations of this study should be acknowledged, as they may have influenced the findings and their interpretation. As a qualitative case study conducted in a single radiology department in 1 hospital in Sweden, the findings are context-specific and not intended to be statistically generalizable. Instead, the value of the study lies in offering in-depth, situated insights that may be transferable to other health care settings facing similar challenges of AI integration.

We used an inductive qualitative approach due to the limited existing knowledge about the experiences of radiologists, radiologic technologists, and other radiology staff regarding the integration of AI applications into radiology practice. We conducted interviews with radiology staff in Sweden to explore these experiences. Participation in the study was entirely voluntary, with interviewees recruited based on their expressed interest in participating. While this approach allowed us to engage individuals motivated to share their experiences, it may also have introduced a selection bias. Specifically, the recruitment strategy may have disproportionately attracted participants who held favorable views toward AI or who were particularly enthusiastic about its integration into radiology practice. As a result, the perspectives captured may lean toward more positive or optimistic attitudes, while voices of those more critical, skeptical, or less engaged with AI technologies may have been underrepresented.

While the sample included radiology staff with diverse experience levels, the relatively small number of participants may not fully capture variations in perspectives across different institutions or health care systems. However, given that many radiology procedures are standardized, the insights gained may still be relevant for hospitals in similar contexts looking to implement AI in radiology.

None of the researchers specialized in radiology; instead, we came from diverse professional backgrounds, presenting both strengths and weaknesses. Notably, none of the interviewers had a background in radiology, which may have limited the depth of probing during the interviews, potentially leading to missed opportunities for deeper exploration of technical or profession-specific issues. Although our varied perspectives could have led to some misunderstandings or missed follow-up questions, this diversity also helped us avoid preconceived notions. The credibility of the study was further enhanced by our multidisciplinary team, which included experts in behavioral economics and systems development (PN), occupational therapy (MN), nursing (IL and PS), health and innovation science (JN), pedagogy (LP), and social and behavioral science (ES). This composition provided multiple perspectives on the issue, combining clinical and scientific expertise, and was reinforced by our significant experience in qualitative research within the health care context. The absence of radiology specialists among the interviewers represents a methodological limitation, as it may have reduced the depth of technical discussion. Conversely, our disciplinary diversity functioned as a strength by promoting open inquiry and minimizing confirmation bias toward existing professional viewpoints.

A total of 12 interviews were conducted, including participants of different professions, ages, levels of experience, and genders, which provided a range of perspectives on radiology practice and AI integration. This diversity provided valuable perspectives on radiology practice. Nevertheless, as the study was conducted within a single Swedish hospital, the contextual specificity limits the transferability of the findings to other institutional or national settings. Most participants had significant hands-on experience with the AI application under study, which added practical depth to the data and supported the study’s credibility [58]. Nevertheless, familiarity with the specific AI tool may have constrained the range of experiences described, as participants may have reflected primarily on 1 particular implementation rather than AI integration more broadly.

It is possible that the participants were generally positive toward AI use, as participants willing to engage in such a study may have had a particular interest in or optimism about technological innovations. This potential self-selection bias should be taken into account when interpreting the findings. While it is possible that participants who volunteered were generally open to technological innovation, the purpose of this study was not to assess attitudes toward AI but to explore professionals’ experiences of working with or around AI technologies. Consequently, the focus was on descriptive accounts of practice rather than on judging AI as positive or negative.

The sample size was considered sufficient based on the concept of information power, which suggests that the more relevant and rich data a sample provides, the fewer participants are needed. Information power is influenced by factors such as the study’s aim, the specificity of the sample, the quality of the interview dialogue, and the depth of analysis [59]. Previous research by Guest et al [60] indicates that 12 interviews are often adequate when participants are knowledgeable about the topic, when the aim is to explore shared experiences rather than compare subgroups, and when the interview material is of high quality. While all participants in our study had relevant radiology knowledge and the interviews adhered closely to the guide, it remains possible that a larger or more heterogeneous sample, including participants from different institutions or with varying levels of exposure to AI, could have uncovered additional themes or perspectives. Quotations from all 12 participants were incorporated into the analysis to enhance transparency and provide a fuller view of the data, but the limited sample still poses a constraint on the transferability of the findings.

### Conclusions

Radiologists, radiologic technologists, and other radiology staff in 1 radiology department in a Swedish hospital experienced many benefits from integrating an AI application into radiology practice, including augmented decision-making, quality of care, and person-centeredness. They also experienced challenges, ranging from preuse adoption to operational hurdles in its routine use in radiology practice. Despite these hurdles, various strategies such as internal training, data refinement, adaptation of work routines, and radiologists’ engagement facilitated the successful integration of AI into radiology practice.

Building on the findings from this case study, we suggest several contextually grounded considerations that may be useful for others seeking to integrate AI into radiology practice. In this particular hospital setting, participants emphasized the importance of structured internal training to ensure staff understood the tools, their purpose, and limitations, often combining formal education with peer learning. Local validation using historical data was seen as vital to confirm that the AI functioned accurately within the specific clinical environment. Adaptations of clinical workflows, including clear responsibilities and follow-up protocols for AI-flagged findings, were described as necessary to make the application work in practice. The role of “super users” or engaged radiologists emerged as influential in fostering staff engagement and maintaining dialogue with vendors. Reliable IT infrastructure and the early involvement of IT staff were also experienced as critical for smooth deployment. Finally, participants stressed that addressing preadoption challenges such as cost, vendor selection, and regulatory processes required proactive planning and stakeholder involvement. While these observations are drawn from a single Swedish hospital and are not intended to be generalizable, they may offer transferable insights for health care organizations navigating similar implementation processes.

## Abbreviations

AI: artificial intelligence
CT: computed tomography
MRI: magnetic resonance imaging
